# Cryptic Myiasis by *Chrysomya bezziana*: A Case Report and Literature Review

**DOI:** 10.4274/tjo.galenos.2020.69360

**Published:** 2020-12-29

**Authors:** Rimpi Rana, Anupam Singh, Sneha Pandurangan, Pratima Gupta, Hemlata Udenia, Ajai Agrawal

**Affiliations:** 1 All India Institute of Medical Sciences, Department of Ophthalmology, Rishikesh, India; 2 All India Institute of Medical Sciences Department of Microbiology, Rishikesh, India

**Keywords:** Anemia, Chrysomya bezziana, ophthalmomyiasis, orbital myiasis

## Abstract

Myiasis is the invasion of living animal tissue by fly larvae. Orbital tissue infestation involvement occurs in 5% of all myiasis cases and is potentially destructive. Infection by *Chrysomya bezziana* is very rare in clinical practice. A 65-year-old woman with history of left eye evisceration presented to the emergency department due to a creeping sensation in the left eye socket and underwent medical and surgical treatment for *C. bezziana* ophthalmomyiasis. A systematic review was performed to identify ophthalmomyiasis cases caused by *C. bezziana* published in PubMed and Embase until December 2019. *C. bezziana* can cause major destruction to both vital and non-vital tissues. It should be treated promptly to prevent extensive damage and life-threatening conditions. This report provides an overview of the epidemiology, causes, risk factors, diagnosis, and treatment options that could assist clinicians in diagnosis and management of this condition.

## Introduction

Myiasis is defined as the infestation of living tissues of humans and other animals by eggs or larvae of flies of the Orthopoda order Diptera. The parasites that most commonly affect the eye and orbit are the larva of *Hypoderma bovis* (hornet fly), Oestrus ovis (sheep botfly), and rarely, *Chrysomya bezziana,* which is an obligate parasite also known as the Old World screwworm.^1^ Orbital involvement occurs in approximately 5% of all the cases of myiasis.^2^ Human myiasis caused by *C. bezziana* was first reported in 1909 in India.^3^ *C. bezziana* myiasis has been largely neglected and is a serious medical condition, though it has not been reported very frequently in humans.^4^ *C. bezziana* infestation differs from typical maggot infestations as it can occur in the absence of existing necrotic tissue and cause extensive damage to living tissue, as in the case reported herein. The condition can even result in death if left undiagnosed.

## Case Report

A 65-year-old housewife presented to the emergency department with complaints of fever along with pain, redness, watering, and swelling of the left upper eyelid for the past 2 days, followed by a crawling sensation with maggots coming out of the socket. Her history included evisceration of the left eye due to perforated corneal ulcer, but there was no history of recent trauma or lesions in the involved area, chronic systemic disease, prolonged use of medications, or progressive loss of weight or appetite.

**Figure 1:**
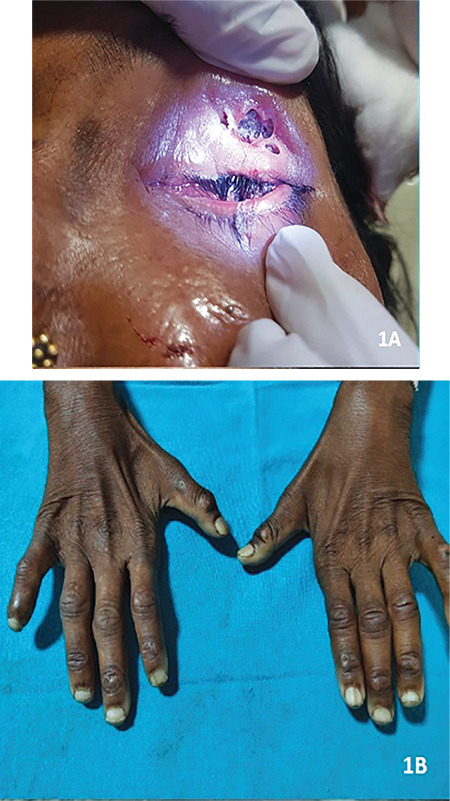
A) Initial presentation with myiasis, B) Swan neck deformity

On examination, the best corrected visual acuity  in her right eye was 20/60. It was pseudophakic with quiet anterior segment and unremarkable posterior segment. On the left side, severe periorbital edema and the eviscerated socket with severe conjunctival congestion and bloody discharge were observed. The upper and lower lids were inflamed and had defects filled with ulcerated necrotic tissue along with blood-stained discharge. Motile white maggots with black fronts were seen crawling in the defects (Figure 1A). They were photosensitive and tried to retract deeper inside in response to light.

On general examination, the patient was emaciated and malnourished (body mass index=18 kg/m^2^). She was well oriented with normal vital signs. On further examination, stiffness of the interphalangeal joints of the hand and feet causing swan neck deformity of the fingers was observed (Figure 1B).

**Figure 2:**
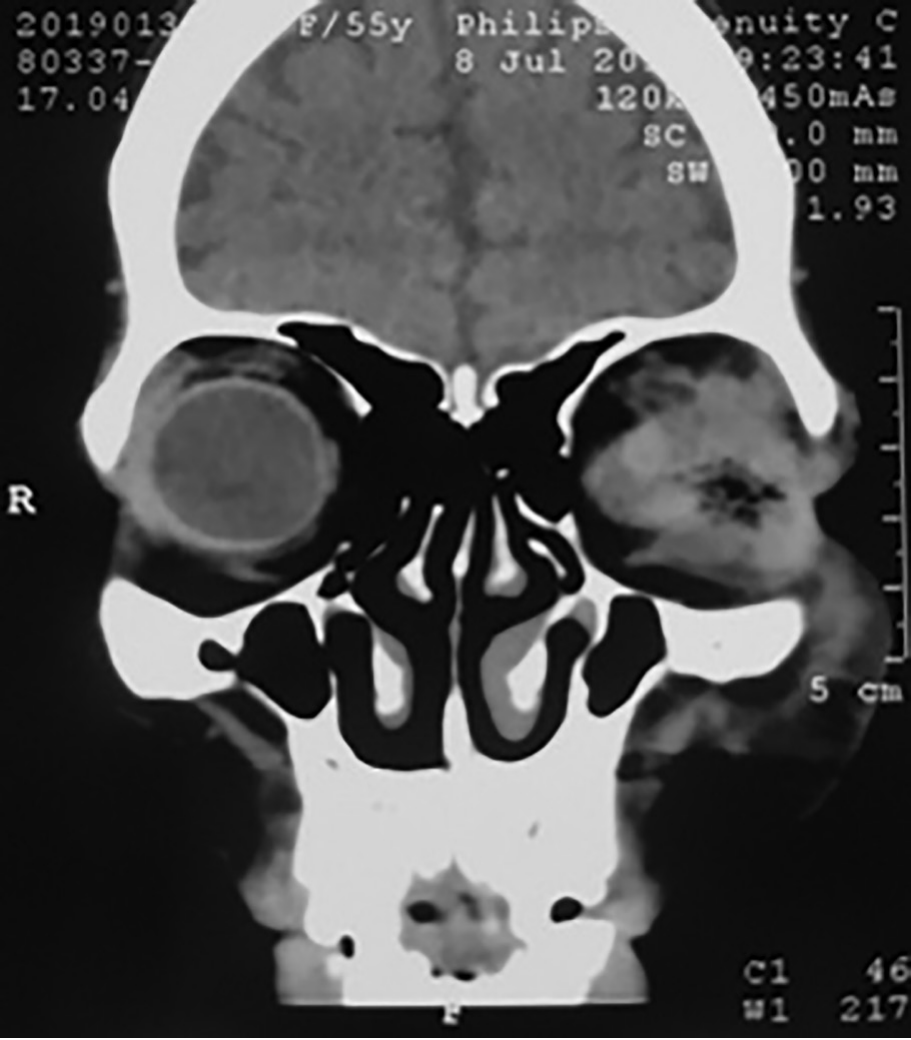
CT scan of the orbit and head CT: Computed tomography

Computed tomography (CT) scan of the paranasal sinuses and orbit was unremarkable (Figure 2). An array of investigations was ordered. Her hemoglobin level was 7.653 g/dL with erythrocyte sedimentation rate of 35 mm/hour. Rheumatoid factor was positive, C-reactive protein (HS) level was 81 mg/dl, and intact parathyroid hormone level was 161 pg/ml. The peripheral blood smear showed normocytic normochromic to microcytic hypochromic with presence of tear drop cells. The rest of the blood reports were unremarkable.

**Figure 3:**
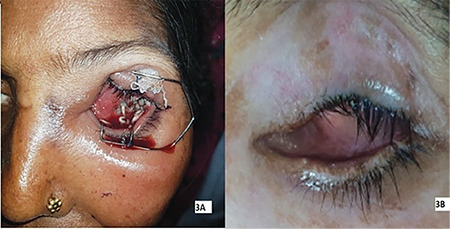
A) Maggots emerging after application of turpentine oil, B) Healed socket after 6 weeks

The patient was diagnosed as having rheumatoid arthritis, microcytic hypochromic anemia with eosinophilia, hypothyroidism, and massive ophthalmomyiasis of the eviscerated socket. She was started on intravenous amoxicillin and clavulanic acid 1.2 g 3 times a day along with oral anti-inflammatory and antacid drugs. Oral albendazole 400 mg once daily was also given for 3 days, which was repeated after 1 week. Considering the wound and sparing of the sinuses and central nervous system on CT scan, topical proparacaine (anesthetic agent) was instilled into the left socket followed by turpentine oil packing, which immobilized the larvae. After 15 to 20 minutes, the larvae were gently removed with forceps (Figure 3A). This procedure was repeated once more. However, some larvae were deeply buried and not amenable to manual removal. Therefore, surgical exploration under local anesthesia was done. Topical moxifloxacin 0.5% drops and ointment were started in the left socket. Wound wash with diluted 3% hydrogen peroxide followed by dressing with 5% povidone-iodine was carried out twice daily under all aseptic precautions. More than 125 larvae were removed in total.

Based on her physical findings and laboratory reports, the patient was started on oral thyroxine sodium (0.25 mg) per day along with multivitamins, hematinic, and protein supplement. On follow-up at 6 weeks, a healed socket (Figure 3B) with improvement in general condition was noted and repeated blood investigations showed a better profile.

**Figure 4:**
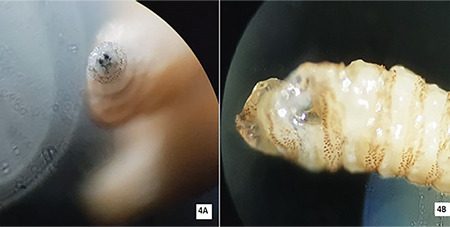
A) Cuticular spines of larva, B) Larva under microscope

A specimen was preserved in 10% formalin and sent to the microbiology department. The larvae were creamy in color with cuticular spines (Figure 4a). They varied in size from 5 to 15 mm due to different stages of presentation. They had strong, robust mouth hooks (Figure 4b), with 4 to 6 papillae on the anterior spiracles. A pigmented dorsal tracheal trunk was noted in the terminal twelfth larval segment. Based on these findings they were confirmed to be larvae of *C. bezziana*.

### Literature Review

A systematic literature review was performed through a search of the PubMed and EMBASE electronic databases to identify all articles related to human orbital myiasis published until December 2019. References from relevant articles were also included. The search strategy was based on an advanced search with the following terms: *“Chrysomya bezziana”* OR “ophthalmomyiasis”, *“Chrysomya bezziana”* AND ophthalmomyiasis, “*C. bezziana*” AND “ophthalmomyiasis”,* “Chrysomya bezziana”* AND orbital myiasis, “*C. bezziana*” AND “orbital myiasis”. Only articles written in English were included. After screening the references, no articles needed to be added.

A total of 204 articles on ophthalmomyiasis in humans were found, in which only 16 cases were reported to be caused by *C. bezziana*. Two case reports had no abstract; information regarding one of these cases was obtained by contacting the author by email and was included in Table 1, and one case was a repetition. On thorough review of the literature, a total of 14 cases attributed to *C. bezziana* were identified (Table 1).^5,6,7,8,9,10,11,12,13,14,15,16,17,18,19^

## Discussion

### Epidemiology

*Chrysomya bezziana* is distributed in about 63 countries in the tropical and subtropical regions of South Asia, Africa, and the Middle East.^20,21^ Cases of ophthalmomyiasis by *C. bezziana* have been observed mainly in India, China (Hong Kong), Iran, Saudi Arabia, Malaysia, and Indonesia, where the climatic conditions are hot and humid.^4^

*C.bezziana* belongs to the order Diptera, family Calliphyridae, and suborder Cyclorrhpha. There are 12 species in the genus Chrysomya. In the literature, most of the species cause myiasis in animals; only *C. bezziana* and *Cochliomyia hominivorax* have been implicated in ophthalmomyiasis in living humans. Humans act as an accidental host, but infections are rarely reported.^22^

### Risk Factors

It is mainly seen with overcrowded conditions, poor sanitation, and poor personal hygiene and in immunocompromised individuals. Chronic debilitating conditions such as diabetes mellitus, fungating carcinomas, psychiatric illness, intellectual disability, hemiplegia, open wounds, use of immunosuppressive agents, poverty, rural background, and neglect may predispose individuals to myiasis.

### Life Cycle

The adult Chrysomya fly is green or blue-green in color. Adult females lay approximately 150–200 eggs at a time on exposed wounds or the mucous membranes of the mouth, ears, and nose. After 24 hours, the eggs hatch and the larvae burrow deep into living tissue in a screw-like fashion, invading host tissues using their sharp mouth-hooks and anchoring with intersegmental spines. The larvae then undergo developmental changes (3 stages of instar) and complete development while feeding on host tissue for 5-7 days. Thereafter, they fall to the ground and pupate, which is temperature-dependent. Sexual maturation occurs in approximately 1-8 weeks. Thus, the life cycle is completed in about 12 weeks.^23,24^

### Presentation

Common presenting features are swelling, itching, ulcer, blood-stained discharge, pain, crawling sensation, and sometimes maggots coming out of the wound. Overall, the presentation varies from minor itching to complete destruction of the globe with apparent myiasis. Early identification and management is very important, as the larvae cause local destruction and inflammation as well as spread deeper into the tissue, potentially extending into the nose, lacrimal gland, paranasal sinuses, and even the brain.

### Diagnosis

Entomological evidence for the species is the gold standard for identification. The larvae are killed by immersion in near boiling water (90-100°C) for 30 seconds before being preserved in 70% to 95% ethanol.^25^ The anatomical features of *C. bezziana* larvae can be used for initial identification: the body shape, body surface with prominent bands of thorn-like spines, papillae, spiracles (posterior and anterior), dorsal tracheal trunks, mouth hooks, and cephalopharyngeal skeleton.^23,24^ Another method is by rearing the larvae to adults for the morphological identification using the adult taxonomic keys.^24^

### Treatment

The larvae exhibit negative phototaxis due to photoreceptors on their anterior end, and they try to move away from light by burying deeper into the tissue. Forceful removal may result in incomplete removal and retention of larval tissue, leading to granulomatous inflammation and calcification.^26^ Therefore, immobilization with ocular paralytics using topical anesthetic agents (cocaine 4-5% solution, lidocaine, pilocarpine 1-4%, proparacaine hydrochloride 0.5%) have been reported to be effective.^27,28^ In spite of paralysis, larvae may adhere to the tissue with their hooks, so various suffocating agents (liquid paraffin, petroleum jelly, beeswax, adhesive tape, pork fat, glue, turpentine oil) and ophthalmic ointments (neomycin, bacitracin, and polymyxin B) are used for successful mechanical removal. Larvicidal agents such as hydrogen peroxide and isopropyl alcohol can also be used. Mechanical removal can be done with the help of jewelers or any other non-toothed forceps under aseptic conditions. Sometimes, the larvae are very deep or damage to the globe is so extensive that mechanical removal is not possible. In such cases, surgical intervention ranging from surgical debridement to complete exenteration of the globe is recommended.

Along with this, the use of systemic broad-spectrum antibiotics such as amoxicillin with clavulanic acid, metronidazole, and cefazolin is indicated to prevent secondary bacterial infections, and antihelminthic drugs such as ivermectin^16^ and benzimidazoles like albendazole and mebendazole are recommended.

It is imperative to treat the underlying cause along with the primary treatment. Cases of orbital myiasis have been reported in immunocompetent patients,^6^ and also in cutaneous malignancies like squamous cell carcinoma^12,13,19^ and basal cell carcinoma.^9,17^ The association with a malignant tumor can be due to the presence of ulcerated and necrotic lesions that are exposed to the environment. The eggs may also be transferred by the patient as a result of scratching.^29^

Myiasis by *C. bezziana* is overall a destructive and rapidly progressing infestation which can also be seen in healthy tissues. For massive ocular myiasis, as reported herein, early intervention is needed to prevent mortality, due to the proximity of the brain and the possibility of intracranial invasion from the orbital apex, which renders this a potentially life-threatening condition. Poor hygiene, rural background, and emaciated condition along with multiple underlying illnesses and neglect were the probable cause of the infection in our patient.

Public awareness of this infestation is needed to encourage personal hygiene and cleanliness. Wound exudates and their odor can attract gravid females to lay eggs on a host, so any open lesions should be kept clean and properly dressed, especially cancerous lesions. Also important is maintaining a clean environment and surroundings with proper disposal of garbage, which attracts flies. Better education and prompt medical services for the community along with improved living conditions are needed for its control.

## Figures and Tables

**Table 1:**
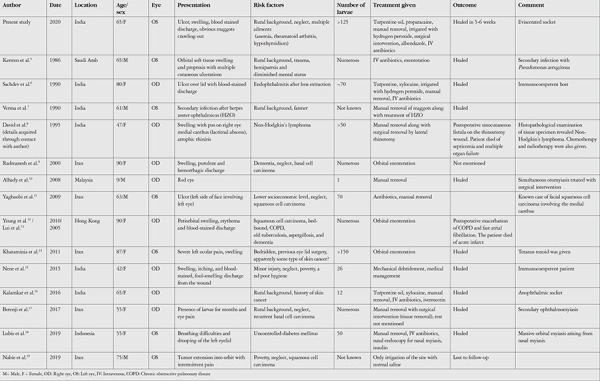
Details of literature included in the review
